# The added-up albumin enhances the diuretic effect of furosemide in patients with hypoalbuminemic chronic kidney disease: a randomized controlled study

**DOI:** 10.1186/1471-2369-13-92

**Published:** 2012-08-29

**Authors:** Bunyong Phakdeekitcharoen, Kochawan Boonyawat

**Affiliations:** 1 Division of Nephrology, Department of Medicine, Faculty of Medicine, Ramathibodi Hospital, Mahidol University, Bangkok, Thailand; 2 Internal Medicine, Department of Medicine, Faculty of Medicine, Ramathibodi Hospital, Mahidol University, Bangkok, Thailand

**Keywords:** Chronic kidney disease (CKD), Furosemide, Albumin, Edema

## Abstract

**Background:**

Chronic kidney disease (CKD) with edema is a common clinical problem resulting from defects in water and solute excretion. Furosemide is the drug of choice for treatment. In theory, good perfusion and albumin are required for the furosemide to be secreted at the tubular lumen. Thus, in the situation of low glomerular filtration rate (GFR) and hypoalbuminemia, the efficacy of furosemide alone might be limited. There has been no study to validate the effectiveness of the combination of furosemide and albumin in this condition.

**Methods:**

We conducted a randomized controlled crossover study to compare the efficacy of diuretics between furosemide alone and the combination of furosemide plus albumin in stable hypoalbuminemic CKD patients by measuring urine output and sodium. The baseline urine output/sodium at 6 and 24 hours were recorded. The increment of urine output/sodium after treatment at 6 and 24 hours were calculated by using post-treatment minus baseline urine output/sodium at the corresponding period.

**Results:**

Twenty-four CKD patients (GFR = 31.0 ± 13.8 mL/min) with hypoalbuminemia (2.98 ± 0.30 g/dL) were enrolled. At 6 hours, there were significant differences in the increment of urine volume (0.47 ± 0.40 *vs* 0.67 ± 0.31 L, *P* < 0.02) and urine sodium (37.5 ± 29.3 *vs* 55.0 ± 26.7 mEq, *P* < 0.01) between treatment with furosemide alone and with furosemide plus albumin. However, at 24 hours, there were no significant differences in the increment of urine volume (0.49 ± 0.47 *vs* 0.59 ± 0.50 L, *P* = 0.46) and urine sodium (65.3 ± 47.5 *vs* 76.1 ± 50.1 mEq, *P* = 0.32) between the two groups.

**Conclusion:**

The combination of furosemide and albumin has a superior short-term efficacy over furosemide alone in enhancing water and sodium diuresis in hypoalbuminemic CKD patients.

**Trial registration:**

The Australian New Zealand Clinical Trials Registration (ANZCTR12611000480987)

## Background

Chronic kidney disease (CKD) is a common problem in clinical practice. The deterioration of renal function impairs salt and water clearance leading to edema and volume overload. The treatment of choice in this situation is administration of a diuretic especially a high potency loop diuretic to enhance free water and salt clearance.

Furosemide is one of the most commonly used high potency loop diuretics in clinical practice. It is an organic acid, which is highly bound to protein (mostly forming furosemide-albumin complex), which reaches the proximal tubular epithelial cells and is secreted in active free form by the anion transporter into the tubular lumen. The site of action of furosemide is the thick ascending limb of the loop of Henle. The mechanism of action is to inhibit active chloride transport at the Na-K-2Cl channel which leads to impaired sodium and chloride reabsorption resulting in natriuresis and free water clearance [1-6].

Despite the high potency of natriuresis, diuretic resistance can still occur. The proposed mechanisms of diuretic resistance are as follows. Firstly, a decline in renal perfusion decreases the rate of drug delivery to its site of action [2,7]. Secondly, severe hypoalbuminemia impairs furosemide secretion into the tubular lumen [8-10]. Thirdly, the accumulation of organic acids, such as hippurate in renal failure state, may compete with furosemide secretion into the tubular lumen via the organic anion transport system at the proximal tubule so decreasing the diuretic activity of furosemide [11-15].

Hypoalbuminemia impairing furosemide secretion was demonstrated by Inoue et al. in 1987 [9]. The studies have shown that analbuminemic rats have a high resistance to furosemide alone compared with the combination of furosemide and albumin which provides more urine output and sodium excretion.

The potential effect of the combination of furosemide and albumin is still controversial. Fliser et al. [16] have found that the effect of the combination of furosemide and albumin can significantly, but modestly increase urine output and sodium excretion in nephrotic syndrome patients. In contrast, others failed to demonstrate this effect [17,18]. In studies of hypoalbuminemic cirrhotic patients, one study has shown the benefit of the combination [19] while others have shown no significant differences in water and sodium excretion between furosemide alone and the combination of furosemide plus albumin [20,21].

Although there is a common usage of the combination of furosemide and albumin in hypoalbuminemic patients in clinical practice, whether there is any significant benefit of this combination for the treatment of edema in hypoalbuminemic patients especially in patients with chronic kidney disease is still unknown. Due to the high price of albumin and allergic reactions which can occur to it [22,23], we conducted a randomized controlled crossover study to compare the efficacy of diuresis between furosemide alone and the combination of furosemide plus albumin for the treatment of edema in stable hypoalbuminemic chronic kidney disease patients by measuring urine output and urine sodium.

### Subjects and methods

#### Subjects

Twenty-four patients with stable chronic kidney disease (no fluctuation of GFR > 10% in two months) who presented with edema (pretibial pitting edema from physical examination) and low serum albumin were enrolled in this study. CKD patients were defined as patients who have GFR < 60 mL/min per 1.73 m^2^ and low serum albumin was defined as serum albumin < 3.5 g/dL. Exclusion criteria were patients with GFR < 10 mL/min per 1.73 m^2^, heavy proteinuria (24-hour urine protein > 3.5 g/d), critically ill patients such as congestive heart failure, acute renal failure, shock, on respirator and/or previous history of furosemide usage within two weeks. The study protocol was approved by the Ethics Committee on Human Studies at Ramathibodi Hospital, Bangkok, Thailand (ID 06-52-18). Written informed consent was obtained from each patient. The study protocol was also registered with the Australian New Zealand Clinical Trials Registration (ANZCTR 12611000480987).

## Methods

**Figure 1:**
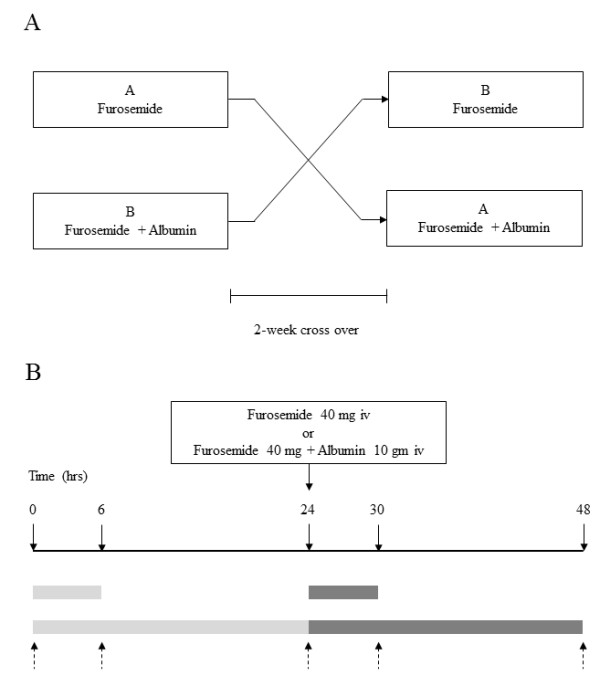
**This scheme shows the steps of the experiment.(A)** Shows diagram of cross over study. **(B)** Shows timeline for blood and urine collections before and after intervention. Pointing down arrow **=** Blood collection, Pointing up arrow = Spot urine collection. Light rectangular box **=** Urine collection before intervention. Dark rectangular box = Urine collection after intervention.

Patient characteristics were documented. Antihypertensive agents, including angiotensin converting enzyme inhibitor (enalapril), angiotensin receptor blocker (candesartan), atenolol, doxazosin and/or hydralazine, were used to control blood pressure. Their dosage was kept unchanged throughout the study. GFR was calculated by the modified diet in renal disease (MDRD) equation (186 × Cr^(−1.154)^ × age^(−0.203)^, and for female multiply by 0.742). Diet with Na = 50 mEq/d and K = 60 mEq/d was designed by a nutritionist and was provided to each patient during the studies. Patients were randomly assigned first to have furosemide alone or the combination of furosemide plus albumin. The body weight, height, and blood pressure were recorded. Blood samples were initially collected from each patient for determination of electrolytes, albumin, blood urea nitrogen and creatinine (at time 0) and then at 6, 24, 30, and 48 hours. Urine was collected to determine the volume and electrolytes at 6 and 24 hours before and after the intervention. Furosemide (40 mg) alone or the combination of furosemide and albumin (10 g of 20% human albumin) was given intravenously at time 24 hours (Figure 1). The batch of salt-poor human albumin used in this study (Zenalb® 20, Bio Products Laboratory, Herts, UK) contained sodium 72 mEq/L (3.6 mEq per 50 ml). Patients were advised to take oral fluids as much as their urine output. After they had completed the first part, they were assigned to have the second part at least two weeks apart (Figure 1). Serum electrolyte, BUN, and creatinine levels were determined using a Technicon Auto Analyzer. The increment of urine output after treatment at each period (6 or 24 hours) was calculated by using post-treatment urine output minus pre-treatment urine output at each corresponding period. Also, the increment of urine sodium was calculated by using the same method.

### Statistical analysis

Statistical analysis was performed using SPSS version 16.0. The mean ± SD was calculated for patient characteristics and diagnostic measures. The number of the sample size was hypothesized based on the power and sample size calculation from the difference of urine output and urine sodium, with α = 0.05 and power = 80%, from a previous study [16]. The calculation revealed 20 patients. The statistical significance of the difference in these measures was examined by using Mann Whitney U- test or *t* test (continuous variable and depends on the data distribution) and chi-square or Fishers’ exact test (categorical variable). The difference was considered significant if the *P*-value < 0.05. For baseline 24-hour urine protein data, they did not show a normal distribution, so we used median and interquartile range for the calculation. A nonparametric test was used to evaluate significant differences between groups.

## Results

Twenty-four stable chronic kidney patients with hypoalbuminemia were included in this study. There were 11 male and 13 female patients. The average GFR in this study was 31.0 ± 13.8 mL/min and the mean serum albumin was 2.98 ± 0.30 g/dL. The other baseline characteristics before intervention have been summarized in Table 1.

**Table 1:** Baseline characteristics of the patients

Numbers	24
Age (years)	66.4 ± 12.8
Males : Females	11 : 13
Weight (kgs)	65.6 ± 11.3
Height (cms)	164 ± 9
BMI (kgs/m^2^)	24.4 ± 4.3
Systolic blood pressure (mmHg)	131.5 ± 6.2
Diastolic blood pressure (mmHg)	79.6 ± 5.5
Serum creatinine (mg/dL)	2.18 ± 0.79
Calculated GFR (mL/min/1.73^2^)	31.0 ± 13.8
Serum albumin (g/dL)	2.98 ± 0.30 (2.32-3.46)
Serum sodium (mEq/L)	139 ± 2.7
Serum potassium (mEq/L)	4.3 ± 0.46
Serum chloride (mEq/L)	104 ± 3.2
Serum bicarbonate (mEq/L)	25.9 ± 3.6
24-hour urine protein (g/d)	0.56^†^ (0.01-3.1)
Causes of chronic kidney disease	Numbers
Hypertension	7
Diabetes mellitus	6
Autosomal dominant polycystic kidney disease	4
Ischemic heart disease	2
Chronic glomerulonephritis	3
Gout	1
Unknown	1

There were no differences in baseline body weight, blood pressure, calculated GFR, serum albumin, urine protein, urine output and urine sodium at 6 and 24 hours between the furosemide alone (F) and the furosemide plus albumin (F + A) group before the intervention (Table 2).

**Table 2:** Baseline parameters before each intervention in chronic kidney disease patients

	**Furosemide**	**Furosemide + Albumin**	***P-*value**
	**Mean ± SD**	**Mean ± SD**	
Weight (kgs)	65.6 ± 11.4	65.7 ± 11.5	0.83
Blood pressure (mmHg) systolic	131.2 ± 6.0	131.8 ± 6.5	0.27
diastolic	79.3 ± 5.3	79.9 ± 5.9	0.33
Calculated GFR (mL/min/1.73^2^)	31.5 ± 14.6	30.5 ± 13.1	0.19
Serum albumin (g/dL)	2.99 ± 0.30	2.97 ± 0.30	0.31
24-hr urine protein (g/d)†	0.45	0.56	0.27††
Urine volume at 6 hours (L)	0.43 ± 0.32	0.40 ± 0.23	0.58
Urine volume at 24 hours (L)	1.95 ± 0.82	1.89 ± 0.59	0.62
Urine sodium at 6 hours (mEq)	17.4 ± 17.7	15.8 ± 13.6	0.32
Urine sodium at 24 hours (mEq)	81.5 ± 46.0	78.3 ± 39.2	0.52
Urine potassium at 6 hours (mEq)	6.89 ± 5.2	6.83 ± 3.99	0.94
Urine potassium at 24 hours (mEq)	37.9 ± 12.2	37.4 ± 13.3	0.87

† Median.

†† Evaluated by nonparametric test.

GFR = Glomerular filtration rate SD = standard deviation.

After the intervention, there were no significant differences in blood pressure and calculated GFR between both groups (Table 3). As expected, there were significant differences in serum albumin at 6 hours (2.98 ± 0.30 *vs* 3.46 ± 0.42 g/dL, *P* < 0.01) and at 24 hours (2.97 ± 0.31 *vs* 3.42 ± 0.41 g/dL, *P* < 0.01) between the furosemide alone and the furosemide plus albumin group (Table 3).

**Table 3:** Comparison of blood pressure, renal function, serum albumin, and urine sodium and potassium after treatment with furosemide alone or with the combination of furosemide plus albumin in chronic kidney disease patients

	**Furosemide**	**Furosemide + Albumin**	***P-*value**
	**Mean ± SD**	**Mean ± SD**	
Blood pressure (mmHg)			
6 hours systolic	132.0 ± 5.1	132.4 ± 6.2	0.60
diastolic	80.1 ± 5.3	80.6 ± 5.3	0.52
24 hours systolic	131.6 ±4.6	132.0 ±5.6	0.60
diastolic	79.7 ± 5.3	80.2 ± 5.5	0.57
Calculated GFR (mL/min/1.73^2^)			
6 hours	27.8 ± 12.6	27.2 ± 10.5	0.64
24 hours	29.1 ± 12.3	28.3 ± 11.3	0.23
Serum albumin (g/dL)			
6 hours	2.98 ± 0.30	3.46 ± 0.42	< 0.01
24 hours	2.97 ± 0.31	3.42 ± 0.41	< 0.01
Urine volume at 6 hours (L)	0.90 ± 0.35	1.07 ± 0.34	0.02
Urine volume at 24 hours (L)	2.44 ± 0.74	2.47 ± 0.60	0.81
Urine sodium at 6 hours (mEq)	54.9 ± 31.6	70.8 ± 31.3	< 0.01
Urine sodium at 24 hours (mEq)	146.8 ± 59.9	154.4 ± 42.2	0.53
Urine potassium at 6 hours (mEq)	14.7 ± 8.0	18.0 ± 8.9	0.03
Urine potassium at 24 hours (mEq)	40.5 ± 10.0	40.3 ± 7.4	0.95

GFR = Glomerular filtration rate SD = standard deviation.

**Figure 2:**
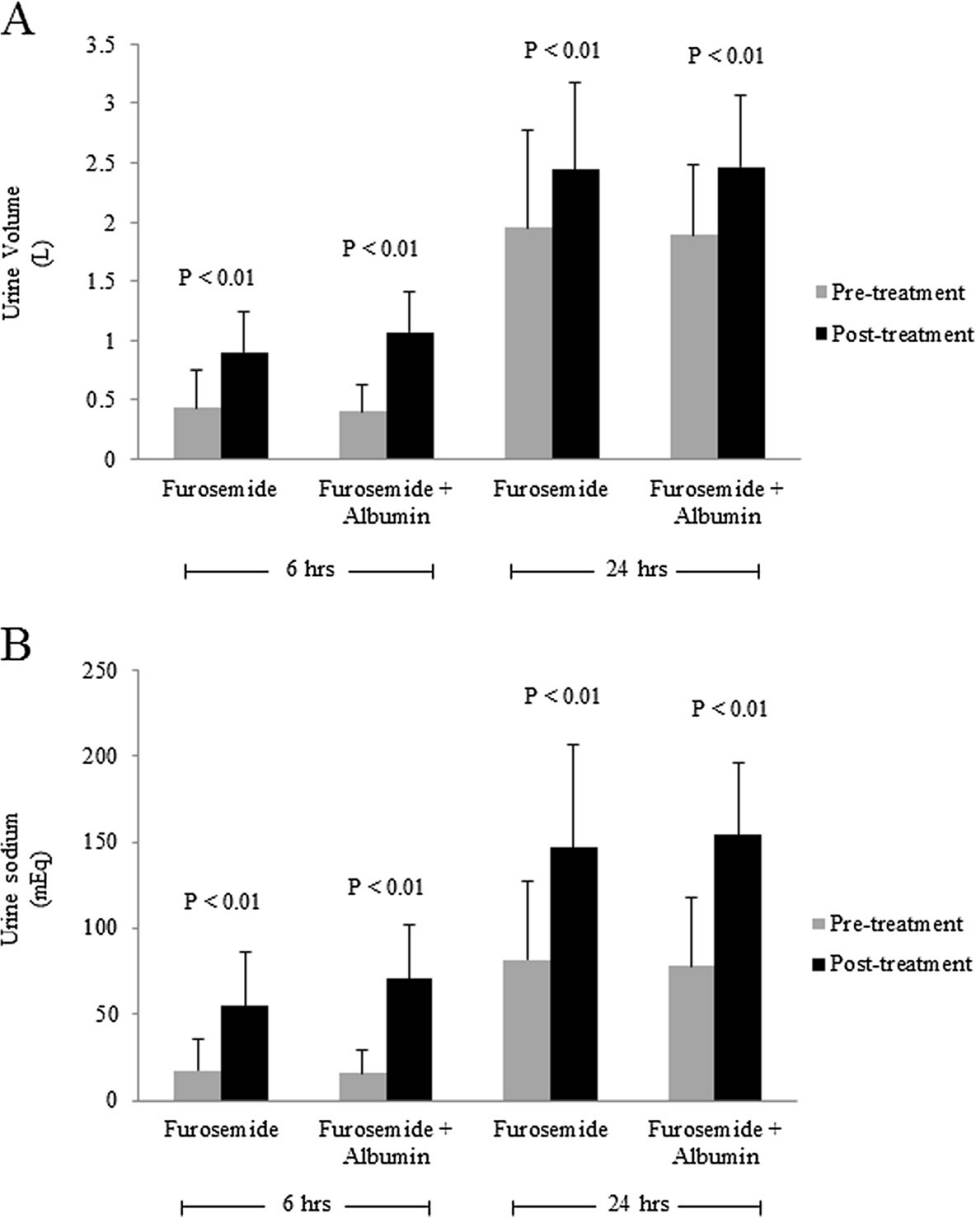
**A.** Comparison of urine volume between pre- and post-treatment with furosemide alone or the combination of furosemide plus albumin in all chronic kidney disease patients. **(B).** Comparison of urine sodium between pre- and post-treatment with furosemide alone or the combination of furosemide plus albumin in all chronic kidney disease patients. Gray box = Pre-treatment, Black box = Post-treatment.

There were significant increases in urine output in post-treatment compared to pre-treatment in both interventions at 6 hours (0.43 ± 0.32 *vs* 0.90 ± 0.35 L, *P* <0.01 for F group, and 0.40 ± 0.23 *vs* 1.07 ± 0.34 L, *P* < 0.01 for F + A group) and at 24 hours (1.95 ± 0.82 *vs* 2.44 ± 0.74 L, *P* <0.01 for F group, and 1.89 ± 0.59 *vs* 2.47 ± 0.60 L, *P* < 0.01 for F + A group) (Figure 2A). Also, there were significant increases in urine sodium in post-treatment compared to pre-treatment in both interventions at 6 hours (17.4 ± 17.7 *vs* 54.9 ± 31.6 mEq, *P* < 0.01 for F group, and 15.8 ± 13.6 *vs* 70.8 ± 31.3 mEq, *P* < 0.01 for F + A group) and at 24 hours (81.5 ± 46.0 *vs* 146.8 ± 59.9 mEq, *P* < 0.01 for F group, and 78.3 ± 39.2 *vs* 154.4 ± 42.2 mEq, *P* < 0.01 for F + A group) (Figure 2B).

**Figure 3:**
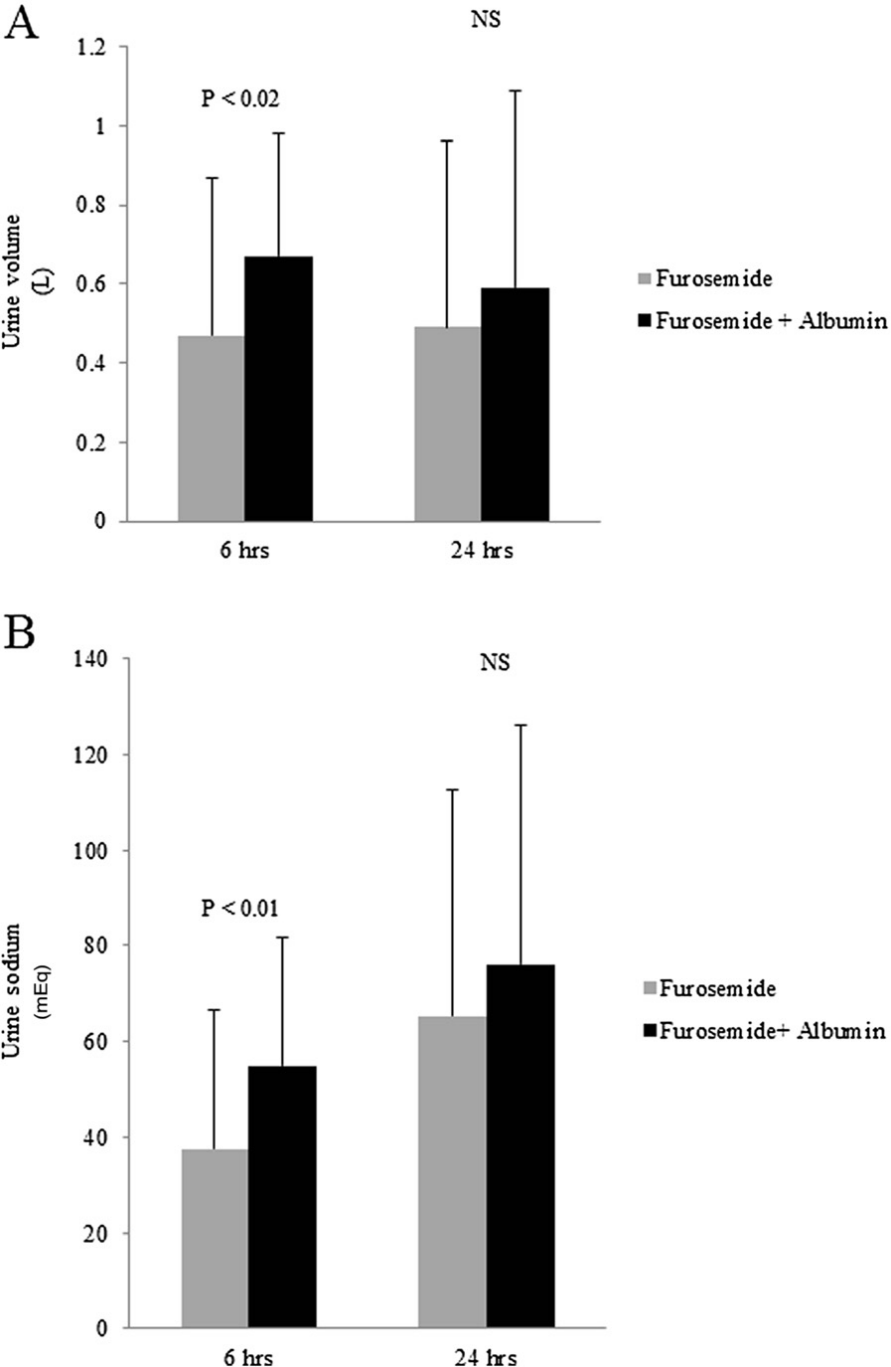
**A.** Comparison of the increment of urine volume after treatment with furosemide alone or with the combination of furosemide plus albumin at 6 and 24 hours in all chronic kidney disease patients. **(B).** Comparison of the increment of urine sodium between after treatment with furosemide alone or with the combination of furosemide plus albumin at 6 and 24 hours in all chronic kidney disease patients. Gray box = Furosemide, Black box= Furosemide + Albumin.

The increments of urine output and urine sodium of each intervention (post-treatment minus pre-treatment urine output/sodium at each corresponding period) were compared (Figure 3). At 6 hours, there were significant differences in the increment of urine volume (0.47 ± 0.40 *vs* 0.67 ± 0.31 L, *P* < 0.02) and urine sodium (37.5 ± 29.3 *vs* 55.0 ± 26.7 mEq, *P* < 0.01) between treatments with furosemide alone and with furosemide plus albumin respectively (Figures 3A and B). However, at 24 hours, there were no significant differences in the increment of urine volume (0.49 ± 0.47 *vs* 0.59 ± 0.50 L, *P* = 0.46) and urine sodium (65.3 ± 47.5 *vs* 76.1 ± 50.1 mEq, *P* = 0.32) between both groups (Figures 3A and B).

After the intervention, there were also significant differences in urine potassium at 6 hours (14.7 ± 8.0 *vs* 18.0 ± 8.9 mEq, *P* = 0.03), but not at 24 hours, between both groups (Table 3).

There were no adverse events such as congestive heart failure, hypertension, or allergic reaction to human albumin/furosemide after both treatments in this study.

## Discussion

In this study, we focused to compare the efficacy of diuretics between furosemide alone and the combination of furosemide plus albumin in stable hypoalbuminemic chronic kidney disease patients with clinical edema and without nephrotic range proteinuria. The results of our study show the short-term beneficial effect (at 6 hours) of the combination of furosemide plus albumin over furosemide alone in natriuresis and diuresis in these patients. This may imply that, in the situation of diuretic resistance, CKD patients with low serum albumin and fluid overload may receive more benefits from this combination regimen.

At 24 hours after treatment, the results of the study did not show the beneficial effect of the combination treatment over furosemide alone. These data are similar to the results studied by Fliser et al. [16]. They found that the superior effect of one single dose of the combination of furosemide plus albumin over furosemide alone is found in only the first 8 hours after treatment. This has also been noted in other studies [24]. This could possibly be due to the short duration of action of furosemide. Furosemide is a short half-life diuretic (1–2 hours) [25,26]. A significant natriuresis and free water clearance is noted during the 6-hour period that the diuretic is acting. However, sodium excretion gradually falls down during the remaining 18 hours of the day, because the associated volume depletion leads to the activation of the sodium-retaining mechanism [27]. Our findings suggest that combination treatment increases the natriuretic potency of furosemide at least at submaximal doses, but does not directly address the issue of whether the same is still true at maximal does of furosemide. Thus, one single dose of the combination of furosemide plus albumin might not be adequate to show the beneficial effect over furosemide alone at 24 hours. Multiple administrations or an increased dose of diuretic may be required to maximize its potency.

Another issue deserving attention may be represented by the finding of a decrease of about 10% in glomerular filtration rate found at 6 hours after treatment in both groups of patients (please compare the calculated GFR finding in Table 2 versus Table 3). This entails that a strictly diuretic strategy in this category of patients (with moderate proteinuria and edema in the absence of oliguria) is encumbered by the risk of inducing a further fall in renal supply and glomerular filtration rate during the action of diuretics. Since we did not have a third study arm with albumin infusion alone, the issue of whether the simple adjunct of intravenous albumin alone is able to induce an increase in diuresis, compared to basal urine volume, in this group of patients cannot be answered.

It should be noted that the rise of serum albumin after human albumin administration in this study is greater than that observed in other studies (Table 3). This could possibly be due to the degree of severity of nephrotic syndrome in most other studies. The amount of proteinuria was high, more than 10 g/d (mean) in some studies [16,17]. After human albumin administration, most of the administrated albumin is likely to be lost in the urine and possibly into the interstitial space. In this study, the median (and range) of proteinuria was 0.5 g/d (0.01-3.1 g/d) which is relatively low compared to other studies. Therefore, it could be expected that the rise of serum albumin in this study should be greater than other studies. Secondly, serum albumin in this study was co-administered with furosemide which caused volume contraction from diuresis. The volume contraction can affect the serum albumin concentration. This can also be observed by the rise of the mean hematocrit of the patients from 32.8 ± 3.2% to 35.8 ± 3.3% (data not shown). Thirdly, our typical Asians’ body weight is much lower than Caucasians’ body weight. In this study, the mean body weight was 65.6 ± 11.4 kgs while the mean body weight of the western population in one study was 90 ± 17.4 kgs [20]. These great differences (2:3) can affect the differences in intravascular volume and the changes in serum albumin concentration after treatment in each population.

The concept of hypoalbuminemia resulting in defective secretion of furosemide has been shown in studies by Inoue et al. [9]. They have demonstrated the mechanism of diuretic resistance in analbuminemic rats (NAR) and hypoalbuminemic patients. The studies have shown that after treatment with furosemide, diuresis significantly increases in normal rats but fails to increase in NAR. However, when administered with both furosemide and albumin, the urine volume in NAR was increased almost three times compared to that of normal rats. They concluded that the binding of furosemide to circulating albumin might be important for the efficient delivery of drugs to the site for their diuretic action. They also demonstrated the efficacy of furosemide and albumin in 16 hypoalbuminemic patients with resistance to furosemide (20–60 mg). The diagnosis of the patients included liver cirrhosis, malignancy and chronic renal failure which had serum albumin ranging from 1.5 – 3.5 g/dL. They concluded that administration of furosemide and albumin enhanced diuresis in NAR, but in hypoalbuminemic patients a well-controlled experimental study should be performed.

Later on, several studies have been performed, mostly in diuretic resistance patients by using a model of patients with nephrotic syndrome and cirrhosis. Fliser et al. [16] studied the efficacy of the combination of furosemide and albumin in 9 patients with nephrotic syndrome. The study was a randomized, double-blind, placebo-controlled, crossover trial. Patients had nephrotic range proteinuria with a mean serum albumin level of 3.0 ± 0.23 g/dL. Most patients had renal function within the normal range (mean GFR of 105 ± 9 mL/min/1.73 m^2^). The results showed modest, but significant increases of cumulative sodium, volume excretion in the combination groups. These results implied that the combination of furosemide and albumin had enhanced diuresis in patients with nephrotic range proteinuria and normal renal function. Another study was performed in 8 nephrotic syndrome patients with serum albumin ranging from 1.1-2.2 g/dL and impaired renal function (serum creatinine 1.2-2.39 mg/dL). The results showed no significant increase in urine volume and sodium excretion in all patients. They did not support the use of albumin in treatment of patients with nephrotic syndrome and supported the mechanism of intratubular albumin that blunts furosemide response [28].

In cirrhotic patients, several studies have demonstrated the efficacy of the combination of furosemide and albumin. Gentilini et al. [19] conducted a randomized, controlled trial to assess the effect of albumin on diuresis response in patients with cirrhosis. The results showed a favourable effect of the combination of these two for the treatment of cirrhotic patients with ascites. They did not mention about the renal function of the patients. Studies by Chalasani et al. [20] in 13 clinically stable cirrhotic patients, with serum creatinine less than 2 mg/dL and 24-hour urine protein less than 100 mg/d, showed a minimal increase in 6-hour urine volume and urine sodium, but there was no statistical significance. A meta-analysis by Rowland et al. [29] has concluded that the effect of the combination of these two is still controversial, but may provide benefit in some selected patients who have recalcitrant edema or ascites or those with severe hypoalbuminemia.

Furosemide is an organic acid, highly bound to plasma protein (91–99%). It is metabolized by uridine diphosphate glucoronyl transferase (UDPGT) in the liver and gut to inactive furosemide glucoronide. Also 85% of the remainder transports to the kidneys. Since furosemide is highly protein-bound, it is not well filtered from the glomerulus. Forty two percent of total furosemide is taken up in the S1 segment of the proximal tubule and metabolized to inactive glucoronide while the remainder is taken up by the S2 segment and is secreted in active form into the lumen by the organic anion transport system [8,10]. The uptake and metabolism by the S1 segment is enhanced by a fall in albumin concentration. Therefore, a low serum albumin concentration enhances furosemide metabolism and leads to decreased tubular secretion of active diuretics [10]. The mechanism of action of furosemide is to inhibit the Na-K-2Cl cotransporter in the luminal membrane of the thick ascending limb of the loop of Henle [1]. Sodium reabsorption takes place via the Na-K-2Cl cotransporter (NKCC). Each molecule of sodium, potassium and chloride binds to NKCC then transformation of NKCC occurs and allows a second molecule of chloride to bind and all of these together will transport into the cell [30]. Furosemide is a competitive inhibitor of the first chloride molecule, thus inhibition of the transformation of the transporter leads to impaired sodium and cation reabsorption in the thick ascending limb of the loop of Henle resulting in natriuresis [30]. The efficacy of furosemide is robust compared to other groups of diuretics. Thus, furosemide is a drug of choice in edematous patients as indicated in acute or chronic kidney disease, congestive heart failure, hepatic cirrhosis and for patients who need natriuresis such as in hypertensive patients [1,31-33].

It is well known that the effect of albumin binding is to trap furosemide in the plasma so it can be delivered to the kidneys as opposed to being distributed throughout the body. In the situation of hypoalbuminemia, less furosemide-albumin complex can be delivered to the kidneys. Moreover, a decline in renal perfusion (CKD in our study) also decreased the rate of drug delivery to its site of action. In addition, a low serum albumin concentration enhanced furosemide metabolism and lead to decreased tubular secretion of active diuretics [10]. Another mechanism of albumin is favouring an enhanced vascular refilling rate which is able to promote effective reabsorption of fluid accumulation from the interstitial space, thereby inducing transient retrieval of increased effective circulating volume. In this manner, an increased renal flow is propitiated and more efficient glomerular filtration function and urine output are achieved irrespective of possible favourable effects on the pharmacodynamics of furosemide. Taken together, our findings in this study are compatible with the hypothesis that the added-up albumin may enhance the rate of furosemide-albumin complex delivery to the kidneys [9], assist the active metabolite of furosemide to its site of action [10] and increase renal blood flow leading to more efficient glomerular filtration function. The effect of increased intratubular albumin which is found in severe nephrotic syndrome patients and which might blunt the response of furosemide was not prominent in our study since all the patients in our studies had 24-hour urine protein less than 3.5 g/d.

## Conclusion

In conclusion, our studies have demonstrated that the combination of furosemide and albumin have a superior short-term efficacy over furosemide alone in enhancing water and sodium diuresis in stable hypoalbuminemic chronic kidney disease patients.

## Abbreviations

CKD: Chronic Kidney Disease; GFR: Glomerular Filtration Rate; MDRD: Modified Diet in Renal Disease.

## Competing interests

The author(s) declare that they have no competing interests.

## Authors’ contribution

BP corresponding author. Conception, design, analysis and interpretation of data. Final approval and revision of the manuscript. KB experiment design, analysis. Interpretation of data. Drafting the article.

## Pre-publication history

The pre-publication history for this paper can be accessed here:

http://www.biomedcentral.com/1471-2369/13/92/prepub
